# Prostate volume and its influence on clinical parameters in prostate cancer detection

**DOI:** 10.1002/jcla.24832

**Published:** 2023-01-04

**Authors:** Carsten Stephan, Robert Peters, Klaus Jung, Andreas Maxeiner

**Affiliations:** ^1^ Department of Urology Charité ‐ Universitätsmedizin Berlin Berlin Germany; ^2^ Berlin Institute for Urologic Research Berlin Germany

To the Editor,

We read with great interest the study by Shan et al.^1^ in detecting prostate cancer (PCa) using clinical parameters based on prostate‐specific antigen (PSA) levels in dependence on prostate volume (PV) and age of patients. The authors compared the diagnostic accuracy of eight parameters: the conventional PSA related parameters total PSA (TPSA), free PSA (FPSA), the ratio of FPSA to TPSA (F/TPSA), the PV‐related parameters (densities) to tPSA and F/TPSA (TPSA/PV = PSAD and (F/TPSA)/PV = (F/T)/PSAD), and corresponding age‐PV related ratios [age of patients/PV = AVR, (age × PV)/TPSA = PSA‐AV and (age × TPSA)/PV = A‐PSAD]. The study was particularly focused on the parameters PSAD, PSA‐AV, A‐PSAD, and AVR that have already been examined in other studies.^2^, ^3^, ^4^, ^5^

In their study group with PV ≤ 30 ml (32 PCa patients and 38 patients suffering from benign prostatic hyperplasia; BPH), all parameters showed a low diagnostic accuracy with AUC values <0.7. On the other hand, in the patient cohort with PV > 30 ml (95 PCa and 277 BPH), the AUC for A‐PSAD (0.736) was significantly (*p* < 0.001) higher than for PSAD (0.709) and all the other PSA‐related parameters, but comparable with AVR (0.742). However, the AUC for AVR did not reach significance compared with PSAD (*p* = 0.176). The authors therefore considered it would be reasonable to validate the clinical utility of AVR and A‐PSAD as cancer screening parameters on more representative cohorts by increasing the sample size.

Therefore, we evaluated AVR and A‐PSAD in a large cohort of 1057 biopsied men (552 PCa and 505 BPH from two German centers),^6^ where the 2010 described Prostate Health Index PHI ([−2]proPSA/freePSA × √PSA) was also available.^7^ As seen in Table 1, PSA‐AV (AUC:0.732) did not improve PSAD (0.726; *p* = 0.08) and A‐PSAD (0.713) had a significant lower AUC than PSAD (*p* < 0.0001). More importantly, PHI (0.801) and PHI derivatives (AUCs from 0.829 to 0.835) always performed better than PSAD, A‐PSAD, and PSA‐AV. In our cohort, the best possible AUC (0.762) was calculated for a logistic regression (LR) model with PSA, age, and PV (LR‐A‐TPSA‐PV, Table 1) but this was still significantly lower than the AUC of PHI.

**TABLE 1:** Diagnostic performance of the different variables calculated by area under the receiver–operating characteristics curves using data of the study by Stephan et al.^6^

Variable	AUC	95% CI	*p* value vs. TPSA	*p* value vs. PSAD	*p* value vs. PHI
TPSA	0.561	0.530–0.591	–	<0.0001; ↓	<0.0001; ↓
FPSA	0.628	0.598–0.657	0.035; ↑	<0.0001; ↓	<0.0001; ↓
F/TPSA	0.753	0.725–0.778	<0.0001; ↑	0.0984; −	0.0020; ↓
PSAD	0.726	0.698–0.753	<0.0001; ↑	–	<0.0001; ↓
(F/T)PSAD	0.511	0.480–0.541	<0.0080; ↓	<0.0001; ↓	<0.0001; ↓
AVR	0.720	0.692–0.753	<0.0001; ↑	0.7037; −	<0.0001; ↓
A‐PSAD	0.713	0.685–0.740	<0.0001; ↑	<0.0001; ↓	<0.0001; ↓
PSA‐AV	0.732	0.704–0.759	<0.0001; ↑	0.0803; −	<0.0001; ↓
LR‐A‐TPSA‐PV	0.762	0.735–0.787	<0.0001; ↑	<0.0004; ↑	0.0187; ↓
PHI	0.801	0.776–0.825	<0.0001; ↑	<0.0001; ↑	–
PHID	0.835	0.811–0.857	<0.0001; ↑	<0.0001; ↑	0.0013; ↑
A‐PHID	0.829	0.805–0.851	<0.0001; ↑	<0.0001; ↑	<0.0001¸↑
PHI‐AV	0.833	0.810–0.855	<0.0001; ↑	<0.0001; ↑	0.0034; ↑
LR‐A‐PHI‐PV	0.835	0.812–0.857	<0.0001; ↑	<0.0001; ↑	<0.0006; ↑

*Note*: ↑ and ↓, increased or decreased value in comparison to the indicated reference value in the table header.

Abbreviations of the variables: (F/T)/PSAD, ratio of F/TPSA to prostate volume; A‐PHID, ratio of (age × PHID) to prostate volume; A‐PSAD, ratio of (age × TPSA) to prostate volume; AVR, ratio of age of patients to prostate volume; CI, confidence interval; F/TPSA, ratio of FPSA to TPSA; FPSA, free PSA; LR‐A‐PHI‐PV, combined variable of age, PHI and prostate volume by logistic regression. Other abbreviations: AUC, area under the receiver–operating characteristics curve; LR‐A‐TPSA‐PV, combined variable of age, TPSA and prostate volume by logistic regression; PHI, prostate health index; PHI‐AV, ratio of (age × prostate volume) to PHID; PHID, ratio of PHI to prostate volume; PSA‐AV, ratio of (age × prostate volume) to TPSA; PSAD, ratio of TPSA to prostate volume; TPSA, total prostate‐specific antigen.

Different prostate volume cutoffs did also not improve the diagnostic performance of PSA‐AV and A‐PSAD as age‐PV‐related parameters in our large cohort. When comparing the AUCs for PV ≤30 and >30 ml, PSA‐AV (AUC: 0.627 vs. 0.704; *p* = 0.13) and A‐PSAD (0.645 vs. 0.677; *p* = 0.543) did not improve, while most other parameters such as PHID (0.709 vs. 0.829; *p* = 0.0179) and PV itself (0.529 vs. 0.706; *p* = 0.0004) were better at PV > 30 ml. The best PV cutoff in our cohort was 48 ml with an equal balance for all parameters and its two AUCs for PV ≤48 and >48 ml.

Additional analysis in summarized data of another large cohort of 1446 biopsied men^8^ and the above‐mentioned prospective cohort (1057 men, sum: 2503 men) showed similar result with no improvement when using PSA‐AV (AUC: 0.699; *p* = 0.0934) in comparison with PSAD (0.696) and a significantly lower AUC for A‐PSAD (0.684; *p* < 0.0001) as compared with PSAD. PHID performed best in the summarized cohort (AUC: 0.784).

We conclude that the PSA‐AV, A‐PSAD, and AVR did not help to improve the diagnostic performance in detecting PCa in large European cohorts up to more than 2500 patients. PHI and PHI‐based parameters always showed the best discrimination power. The effect in the smaller Chinese cohort in the study by Shan et al.^1^ might be based on prostate volume differences or simply on the sample size.

## AUTHOR CONTRIBUTIONS

All authors contributed equally to this work.

## CONFLICT OF INTEREST

All authors declare no conflict of interest.

## Data Availability

The data used to support the findings of this report are included in the article.
